# Lipid membrane composition modulates the structural evolution of early‐stage human islet amyloid polypeptide amyloids

**DOI:** 10.1002/pro.70867

**Published:** 2026-11-05

**Authors:** Daniel Warren, Jadon Sitton, Joshua Skrehot, Dmitry Kurouski

**Affiliations:** ^1^ Department of Biochemistry and Biophysics Texas A&M University College Station Texas USA; ^2^ Department of Biomedical Engineering Texas A&M University College Station Texas USA

**Keywords:** AFM‐IR, fibrils, hIAPP, oligomers

## Abstract

Progressive β cell death resulting from the aggregation of human islet amyloid polypeptide (hIAPP) in the pancreas is a molecular driver of type 2 diabetes (T2D). Recent studies from our group have shown that hIAPP forms two distinct amyloid oligomers: on‐path donut oligomers (DOs) and off‐path round oligomers (ROs). Previous studies have also shown lipids, one of the most abundant classes of biomolecules within cells, alter the kinetics of hIAPP amyloid formation and template structurally distinct hIAPP fibrils. However, the influence of the specific lipid environment on the evolution of early‐stage hIAPP amyloids remains unknown. In this study, we employ atomic force microscopy (AFM) and atomic force microscopy Infrared (AFM‐IR) spectroscopy to resolve the morphological and structural dynamics of early‐stage hIAPP DOs, ROs, and fibrils (FBs) formed in the presence of phosphatidylcholine (PC) or phosphatidylserine (PS). PC‐templated DOs with predominantly parallel β‐sheet structure, while PS‐templated DOs were predominantly antiparallel β‐sheet. These DOs were present only during the early stages of aggregation. Conversely, ROs were persistent and their structural evolution was more stochastic irrespective of the lipid environment. The secondary structure of lipid‐templated DOs correlated precisely with that of early‐stage FBs, highlighting their role as on‐path oligomers in hIAPP aggregation. Finally, the lipid environment present during aggregation uniquely altered the structural evolution of FBs, with PC favoring a progressive increase in antiparallel β‐sheet FBs and PS favoring an increase in parallel β‐sheet. These results provide crucial insight into the mechanisms of hIAPP aggregation in the lipid‐rich cellular environment, simultaneously solidifying lipids as critical players in the early stages of hIAPP aggregation.

## 1 INTRODUCTION

The aggregation of human islet amyloid polypeptide (hIAPP) is a hallmark feature of type 2 diabetes (T2D), a debilitating disease affecting over 520 million people globally (Collaborators, G. B. D. D, 2023; Hassan et al., 2022). Present in approximately 90% of cases, hIAPP amyloid oligomers and fibrils (FBs) accumulate within the pancreatic islets and elicit high cytotoxicity to β cells (Asthana et al., 2018; Kanatsuka et al., 2018; Westermark & Grimelius, 1973). The loss of such cells leads to insulin insufficiency and a state of chronic hyperglycemia, accelerating T2D pathology and its associated comorbidities (Giri et al., 2018; Laakso, 1999). Additionally, hIAPP amyloids have been observed to cross‐seed the aggregation of Amyloid β (Aβ) and α‐synuclein (α‐syn), the primary constituents of amyloid plaques in Alzheimer's and Parkinson's diseases, respectively (Meng et al., 2023; Moreno‐Gonzalez et al., 2017; Mucibabic et al., 2020).

Recent studies from our group have demonstrated that aggregation of hIAPP results in a heterogenous population of two structurally and morphologically distinct amyloid oligomers (Warren et al., 2026). Specifically, parallel β‐sheet rich donut oligomers (DOs) and anti‐parallel β‐sheet rich round oligomers (RO) were both formed in the earlier stages of hIAPP aggregation. Additionally, it was observed that DOs were no longer present after 4 h of hIAPP aggregation, while ROs remained persistent after 24 h of aggregation. It was also observed that DOs structural evolution matched that of FBs before DO populations diminished and FBs became dominant. Together this suggests that DOs are “on‐path” oligomers which evolve into parallel β‐sheet rich FBs while ROs are “off‐path” persistent oligomers. Thus, RO do not propagate into fibrils.

Simultaneously, it has been reported that lipids drastically alter the aggregation of hIAPP (Brender et al., 2012; Sciacca et al., 2016; Sciacca et al., 2020; Soong et al., 2009). Nanga and colleagues reported that hIAPP interacts with membranes, which induces the formation of a helical intermediate that accelerates aggregation (Nanga et al., 2011). Sitton and colleagues found that saturated phospholipids and sphingolipids accelerated hIAPP aggregation, and the extent to which this accelerating effect occurred correlated with lipid headgroup charge (Sitton et al., 2025). The presence of lipid membranes during aggregation simultaneously templated morphologically and structurally distinct fibril polymorphs. Similar findings were reported by Sitton and coworkers for hIAPP aggregation in the presence of fatty acids, with the length and saturation of the fatty acids modulating the aggregation rate of hIAPP and templating unique fibril polymorphs (Sitton et al., 2026). Together, these results indicate that lipids catalyze hIAPP aggregation, and the chemical properties of the lipid environment determine the rate of hIAPP aggregation and resulting fibril structure.

Building upon these findings, this study aims to elucidate how lipids alter the structural evolution of hIAPP DOs, FBs, and ROs during early stages of aggregation. Here, we utilize monolipid large unilamellar vesicles (LUVs) composed of 1,2‐dipalmitoyl‐*sn*‐glycero‐3‐phospho‐l‐serine (DPPS) and 1,2‐dipalmitoyl‐*sn*‐glycero‐3‐phosphocholine (DPPC) as model membranes. We then aggregate hIAPP in the presence of LUVs under physiological conditions (37°C, pH 7.4) and collect samples at different time points (0.5, 1, 2, 16 h) for analysis via atomic force microscopy (AFM) and atomic force microscopy Infrared (AFM‐IR) spectroscopy (Centrone, 2015; Katzenmeyer et al., 2013; Lahiri et al., 2013; Ramer et al., 2018; Ruggeri, Flagmeier, et al., 2020a; Ruggeri, Mannini, et al., 2020b). While AFM is capable of visualizing the morphology of protein aggregates present in the sample, AFM‐IR can be used to probe the secondary structure of individual oligomers and fibrils (Banerjee & Ghosh, 2021; Ruggeri et al., 2015; Ruggeri et al., 2018). For this, the scanning probe used in AFM‐IR can be placed on such oligomers (Dazzi et al., 2012; Dazzi & Prater, 2017). Next, pulsed tunable IR light is used to trigger thermal expansions that are reordered by the scanning probe and transformed into IR spectra. Finally, the amide I band in such spectra is analyzed to determine the secondary structure of protein specimens. Recently, AFM‐IR has been used to probe the secondary structure of hIAPP (Warren et al., 2026), α‐syn (Zhou & Kurouski, 2020), Aβ (Zhaliazka & Kurouski, 2022), TDP‐43 (Pickett et al., 2026), and Tau (Skrehot et al., 2026) oligomers formed in the absence of lipids. However, the molecular mechanisms of lipid‐driven aggregation of amyloidogenic proteins remain unclear. To bridge this gap, we utilize AFM‐IR to reveal the mechanisms of hIAPP aggregation in the lipid‐rich cellular environment.

## 2 RESULTS

### 2.1 Aggregation in the presence of DPPC and DPPS uniquely alter the secondary structure and morphology of early‐stage DOs, FBs, and ROs

To investigate how a lipid environment influences hIAPP aggregation, we first examined the work of Sitton and co‐workers, who showed that hIAPP aggregates more rapidly in the presence of equimolar DPPC or DPPS than in a lipid‐free environment. In their study, DPPS shortened *t*_lag_ to 1.08 ± 0.06 h and *t*_half_ to 1.62 ± 0.1 h, while DPPC shortened *t*_lag_ to 1.38 ± 0.03 h and *t*_half_ to 2.07 ± 0.08 h (Sitton et al., 2025). The stronger accelerating effect of DPPS compared to that of DPPC is consistent with the electrostatic interaction between the negatively charged serine head group and the positively charged residues of hIAPP, which facilitates nucleation at the membrane surface and promotes rapid aggregation (Skeby et al., 2016). These kinetic results indicate that hIAPP quickly forms aggregates in both lipid environments, motivating our investigation of early time points (0.5 and 1 h) to capture the morphology and structure of the oligomers directly involved in fibril propagation. We also investigated 2 and 16 h aggregate species to track evolution throughout fibril maturation.

To resolve the effect of each lipid on the secondary structure of individual hIAPP aggregate species, we utilized AFM‐IR Spectroscopy to analyze DOs at 1 h, ROs at 2 h, and FBs at 1 h in both the hIAPP:PC and hIAPP:PS conditions. These time points were selected to capture each species at a stage where they were consistently present across both conditions. Gaussian peak fitting of the amide I band was performed to quantify relative concentrations of respective secondary structures.

#### 2.1.1 Donut oligomers

AFM‐IR analysis of hIAPP DOs formed after 1 h of aggregation in the presence of DPPC revealed a secondary structure composed of 27.9% ± 1.0% parallel β‐sheet, 21.4% ± 0.7% anti‐parallel β‐sheet, 1.9% ± 0.1% random coil, and 48.75% ± 1.3% β‐turn/α‐helix (Figure 1a,c). In contrast, hIAPP:PS DOs formed after 1 h of aggregation yielded an average secondary structure composition of 18.6% ± 2.2% parallel β‐sheet, 31.5% ± 4.1% anti‐parallel β‐sheet, 26.4% ± 4.3% random coil, and 23.6% ± 2.8% β‐turn/α‐helix (Figure 1b,c). Thus, while both lipid environments support the formation of β‐sheet rich DO, aggregates formed in DPPS favored a higher antiparallel β‐sheet and random coil content, whereas aggregates formed in DPPC favored a more β‐turn rich conformation. These lipid‐specific findings are consistent with findings of Dou and coworkers, who demonstrated that DPPC and DPPS each uniquely altered the secondary structure of α‐synuclein oligomers (Dou & Kurouski, 2022). We also observed that hIAPP:PS DOs formed after 1 h of aggregation were significantly thicker (8.25 ± 2.04 nm) than DOs formed in the presence of DPPC (6.56 ± 1.91 nm) (Figure 1d–f). Together, these results indicate that the composition of the lipid environment present during early stages of aggregation determines the polymorphic properties of early stage hIAPP DOs.

**FIGURE 1:**
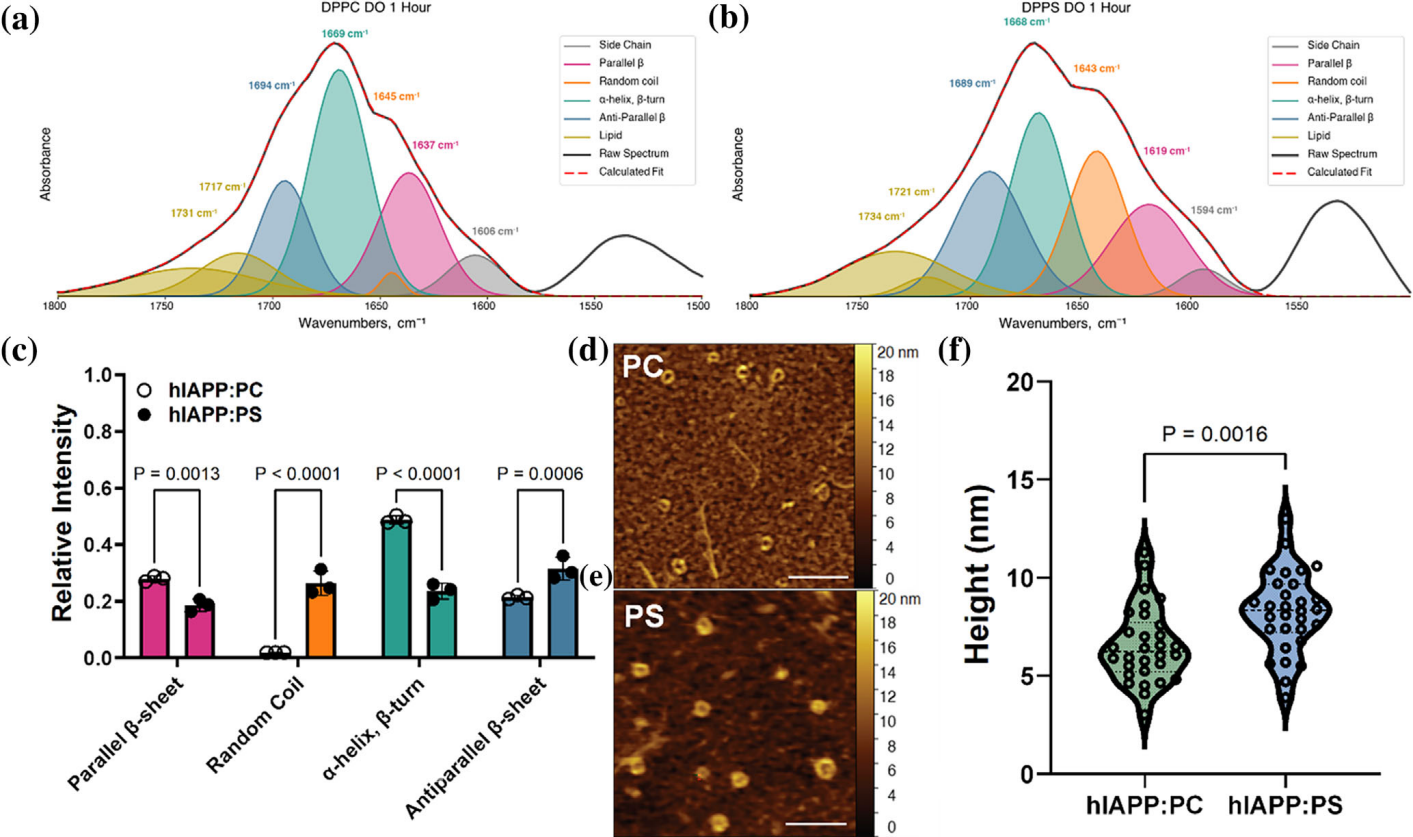
Representative Gaussian peak‐fitted AFM‐IR spectra obtained from hIAPP donut oligomers (DOs) formed in the presence of (a) DPPC or (b) DPPS at a 1:1 protein:lipid molar ratio after 1 h of aggregation. (c) Quantification of relative secondary structure content from *n* = 3 DOs peak‐fitted spectra per condition. Representative 2.0 × 2.0 μm AFM images of hIAPP DOs formed in the presence of (d) DPPC or (e) DPPS after 1 h of aggregation. (f) Quantification of *n* = 30 DO profile height measurements from AFM images. Image scale bars = 500 nm. Statistical testing performed using two‐way ANOVA with Tukey's post hoc test (c) or Welch's *t*‐test (f).

#### 2.1.2 Fibrils

AFM‐IR analysis of hIAPP FBs formed after 1 h of aggregation in the presence of DPPC were comprised of 31.8% ± 2.9% parallel β‐sheet, 27.9% ± 2.3% anti‐parallel β‐sheet, 2.1% ± 0.1% random coil, and 38.2% ± 1.1% β‐turn/α‐helix (Figure 2a,c). hIAPP:PS FBs formed after 1 h contained 34.7% ± 9.0% parallel β‐sheet, 34.6% ± 2.2% anti‐parallel β‐sheet, 2.5% ± 2.5% random coil, and 28.2% ± 5.3% β‐turn/α‐helix (Figure 2b,c). Comparatively, FBs formed in both lipid environments are dominated by β‐sheet conformations. However, the presence of DPPS during early stages of aggregation significantly decreases the amount of β‐turn/α‐helix content relative to hIAPP:PC 1 h FBs. Additionally, we observed the thickness of hIAPP:PS FBs formed after 1 h was significantly thicker (6.05 ± 1.32 nm) than those formed in the presence of DPPC (5.06 ± 1.41 nm) (Figure 2d–f). These lipid‐dependent differences demonstrate that lipid membranes template not only unique early oligomeric polymorphs, but also the fibrillar species that present during early stages of aggregation.

**FIGURE 2:**
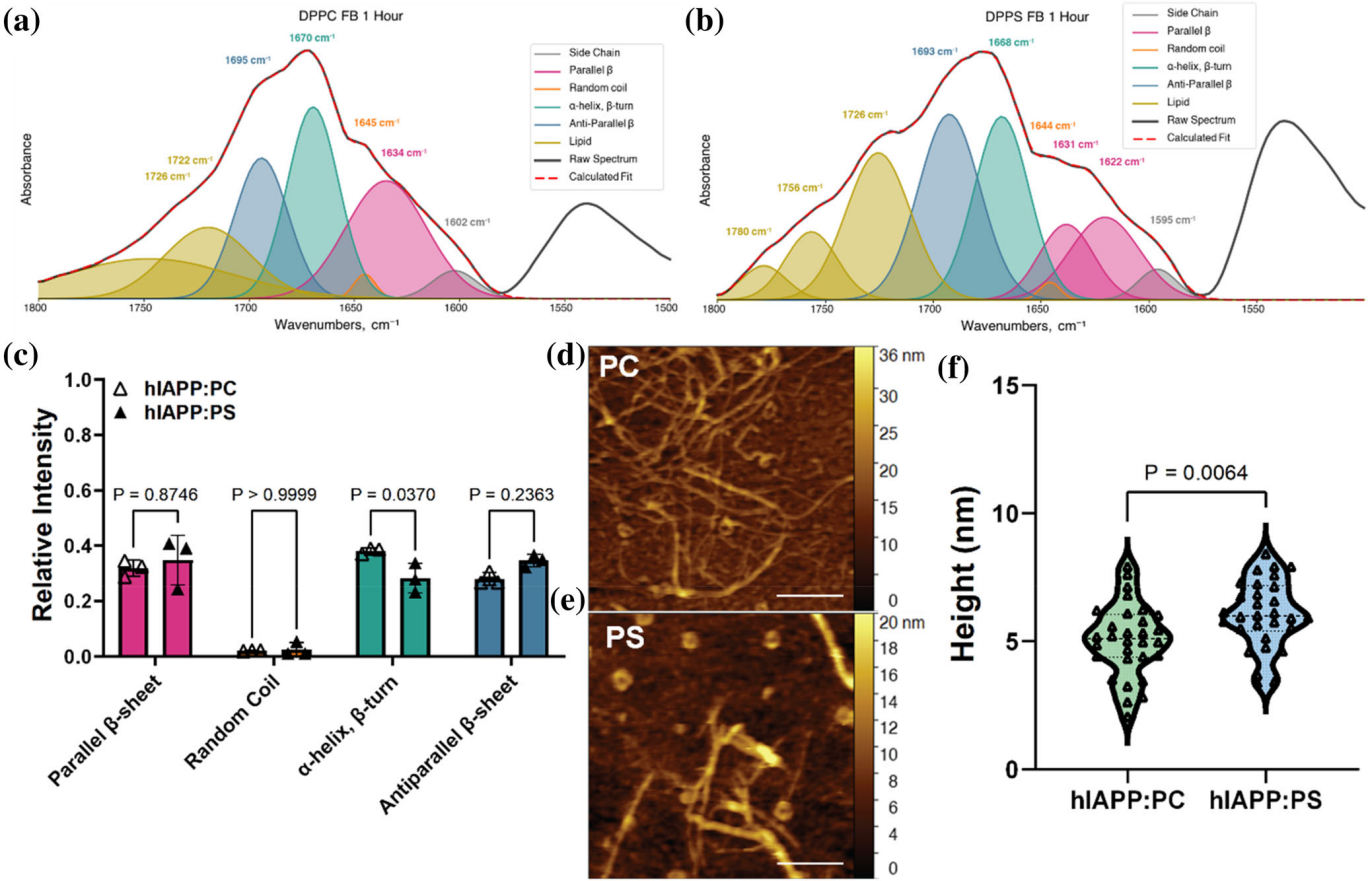
Representative Gaussian peak‐fitted AFM‐IR spectra obtained from hIAPP fibrils (FBs) formed in the presence of (a) DPPC or (b) DPPS at a 1:1 protein:lipid molar ratio after 1 h of aggregation. (c) Quantification of relative secondary structure content from *n* = 3 FBs peak‐fitted spectra per condition. Representative 2.0 × 2.0 μm AFM images of hIAPP FBs formed in the presence of (d) DPPC or (e) DPPS after 1 h of aggregation. (f) Quantification of *n* = 30 FB profile height measurements from AFM images. Image scale bars = 500 nm. Statistical testing performed using two‐way ANOVA with Tukey's post hoc test (c) or Welch's *t*‐test (f).

#### 2.1.3 Round oligomers

AFM‐IR analysis of hIAPP ROs formed after 2 h in the presence of DPPC revealed a secondary structure consisting of 22.0% ± 11.0% parallel β‐sheet, 27.34% ± 5.33% anti‐parallel β‐sheet, 9% ± 9% random coil, and 41.7% ± 8.5% β‐turn/α‐helix (Figure 3a,c). hIAPP:PS ROs formed after 2 h were also β‐sheet rich but structurally distinct, containing 19.3% ± 9.8% parallel β‐sheet and 16.8% ± 12.3% random coil, in addition to significantly less β‐turn/α‐helix content (8.5% ± 4.5%) and significantly greater anti‐parallel β‐sheet content (55.5% ± 2.1%) (Figure 3b,c). Early‐stage hIAPP:PS ROs were also significantly smaller (6.66 ± 1.39 nm) compared to hIAPP:PC ROs formed over the same length of time (10.69 ± 2.84 nm). These data show that even at the same aggregation stage, the RO formed in the presence of DPPC and DPPS have characteristically distinct morphologies and structural compositions, highlighting the modulatory effect of lipid membrane chemistry on oligomer structure.

**FIGURE 3:**
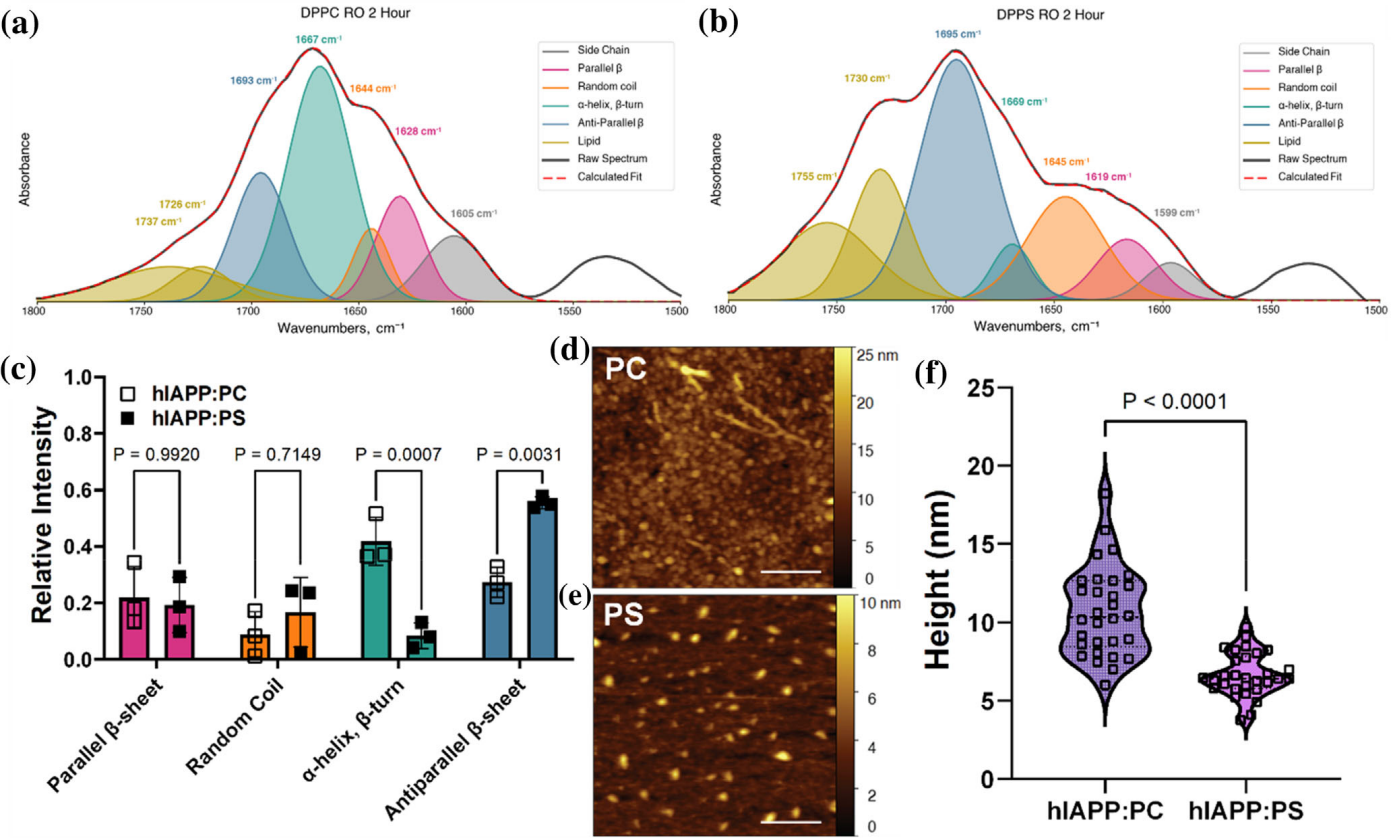
Representative Gaussian peak‐fitted AFM‐IR spectra obtained from hIAPP round oligomers (ROs) formed in the presence of (a) DPPC or (b) DPPS at a 1:1 protein:lipid molar ratio after 1 h of aggregation. (c) Quantification of relative secondary structure content from *n* = 3 ROs peak‐fitted spectra per condition. Representative 2.0 × 2.0 μm AFM images of hIAPP ROs formed in the presence of (D) DPPC or (e) DPPS after 1 h of aggregation. (f) Quantification of *n* = 30 RO profile height measurements from AFM images. Image scale bars = 500 nm. Statistical testing performed using two‐way ANOVA with Tukey's post hoc test (c) or Welch's *t*‐test (f).

### 2.2 Nanoscale analysis of hIAPP aggregate morphology and secondary structure at different stages of aggregation in the presence of DPPC and DPPS reveal distinct secondary structure evolution

We next examined how DO, FB, and RO morphologies and secondary structures evolved over the full course of hIAPP aggregation. For each lipid condition, AFM images and AFM‐IR spectra were collected from aggregate species found at 0.5, 1, 2, and 16 h of aggregation.

#### 2.2.1 Evolution of donut‐like oligomers

The observed hIAPP DOs formed in the presence of DPPC after 0.5 h exhibited a mean thickness of 7.56 ± 1.65 nm an evenly distributed secondary structure content, with 28.6% ± 1.1% parallel β‐sheet, 24.5% ± 1.4% anti‐parallel β‐sheet, 24.6% ± 1.7% random coil, and 22.4% ± 2.0% β‐turn/α‐helix (Figure 4a,c,d). Although the DO thickness did not significantly change at 1 h, hIAPP:PC DOs converted to a more ordered, significantly more β‐turn rich makeup (28.0% ± 1.0% parallel β‐sheet, 21.4% ± 0.7% anti‐parallel β‐sheet, 1.9% ± 0.1% random coil, and 48.8% ± 1.3% β‐turn/α‐helix). This indicates that in the presence of DPPC, hIAPP DOs decrease in disordered content and consolidation of β‐turn and β‐sheet makeup over time. DOs were not observed at 2 or 16 h, consistent with their role as transient “on‐path” aggregates (Warren et al., 2026).

**FIGURE 4:**
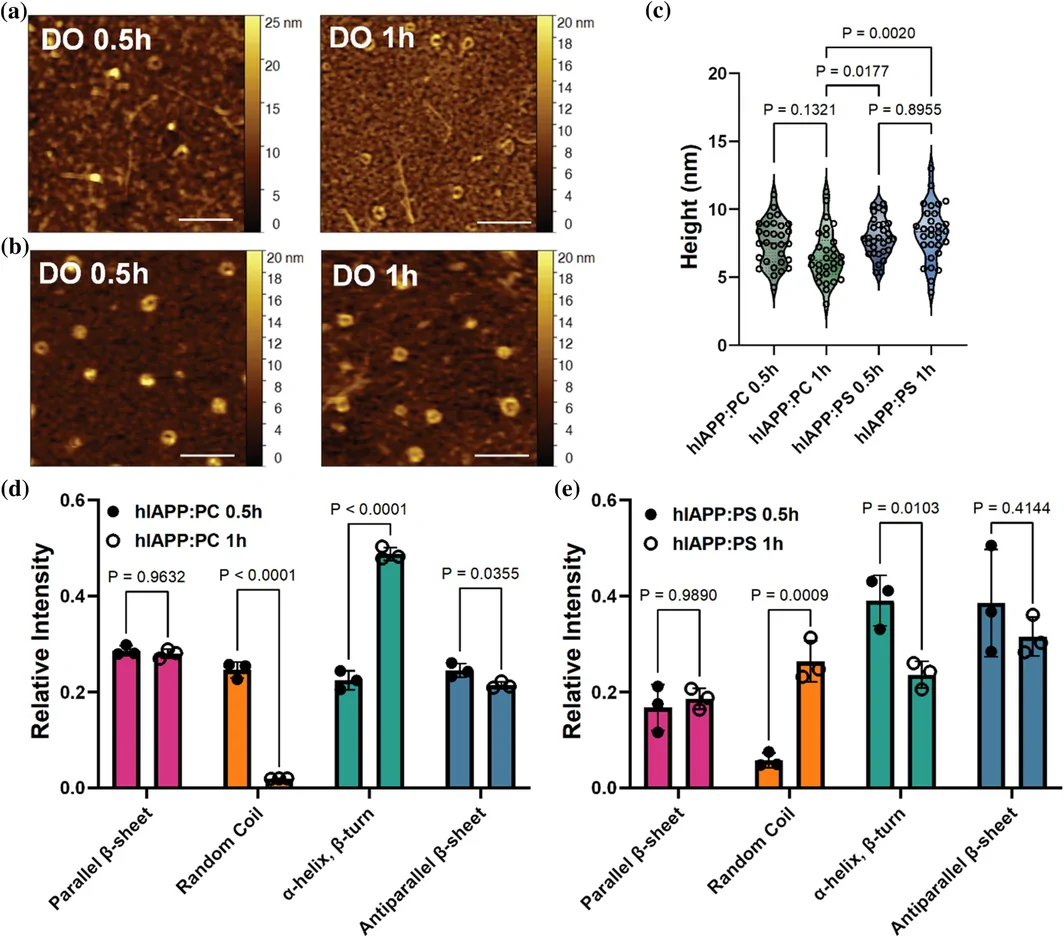
Representative 2.0 × 2.0 μm AFM images of hIAPP donut oligomers (DOs) formed in the presence of (a) DPPC or (b) DPPS LUVs at a 1:1 protein:lipid molar ratio after 0.5 or 1 h of aggregation. (b) Quantification of profile height from *n* = 30 DOs per condition. Quantification of relative secondary structure content Gaussian peak fitting of *n* = 3 DO AFM‐IR spectra per condition. Image scale bars = 500 nm. Statistical testing performed using two‐way ANOVA with Tukey's post hoc test.

In contrast, hIAPP:PS DOs formed after 0.5 h were 7.93 ± 1.41 nm thick and contained a high makeup of β‐sheet and β‐turn structure (16.7% ± 4.8% parallel β‐sheet, 38.5% ± 11.2% anti‐parallel β‐sheet, 5.7% ± 1.6% random coil, and 39.0% ± 5.3% β‐turn/α‐helix) (Figure 4b,c,e). Similar to hIAPP:PC DOs, the aggregate thickness did not significantly change at 1 h of aggregation. However, hIAPP:PS DOs conformationally changed to a more random coil‐rich structure (18.6% ± 2.19% parallel β‐sheet, 31.5% ± 4.04% anti‐parallel β‐sheet, 26.4% ± 4.3% random coil, and 23.6% ± 2.8% β‐turn/α‐helix). It is important to note that hIAPP:PS DOs were significantly thicker than those formed in the presence of DPPC after 1 h of aggregation. Akin to what was observed for hIAPP:PC DOs, DOs were not detected at 2 or 16 h of aggregation in the presence of DPPS. Together, these data demonstrate that while DOs are rapidly transient intermediates in both lipid environments, DPPC drives them toward a more β‐turn rich conformation, while DPPS favors a more mixed β‐sheet and random coil makeup. This suggests that lipids not only accelerate DO formation and conformational changes but also template unique hIAPP DO polymorphs.

Our findings also show that the presence of lipid membranes substantially compacts their physical dimensions and accelerates DOs' clearance relative to lipid‐free conditions. In the absence of lipids, DOs measure 17.48±2.78nm at 1 h, thinning to 13.78±2.47nm at 4 h before disappearing completely by 8 h; structurally, they transition from an equal split of parallel and antiparallel β‐sheets (~40% each with ~20% disordered) to a parallel β‐sheet‐dominated conformation (~60% parallel β‐sheet and <10% disordered) that matches mature fibril architecture (Warren et al., 2026). In contrast, both DPPC and DPPS compress DO heights to ~6.5–8.3 nm and compress their lifespan to under 2 h, leaving them undetectable by 2 and 16 h. Despite sharing this rapid clearance, the two lipids steer DO secondary structure along divergent paths: DPPC drives an initially balanced distribution (28.6% parallel β‐sheet, 24.5% antiparallel β‐sheet, 24.6% random coil, and 22.4% α/β‐turn) into an ordered β‐turn/α‐helix‐rich form by 1 h (48.8% α/β‐turn, 28.0% parallel β‐sheet, and 1.9% random coil). Conversely, DPPS templates DOs that are thicker (8.25±2.04nm at 1 h) and evolve from an initial β‐rich state (38.5% antiparallel β‐sheet, 39.0% α/β‐turn, and 16.7% parallel β‐sheet) into an antiparallel β‐sheet and random coil‐rich conformation (31.5% antiparallel β‐sheet, 26.4% random coil, 23.6% α/β‐turn, and 18.6% parallel β‐sheet).

#### 2.2.2 Evolution of fibrils

After 0.5 h of aggregation in the presence of DPPC, hIAPP:DPPC FBs were observed to have a mixed, β‐turn and β‐sheet rich secondary structure content (25.6% ± 2.9% parallel β‐sheet, 22.2% ± 1.5% antiparallel β‐sheet, 2.8% ± 0.2% random coil, 49.3% ± 2.2% β‐turn/α‐helix) and a mean thickness of 6.49 ± 2.42 nm (Figure 5a,c,d). After 1 h, DPPC‐templated FBs showed a significant decrease in β‐turn/α‐helix content (31.8% ± 2.9% parallel β‐sheet, 27.9% ± 2.3% antiparallel β‐sheet, 2.1% ± 0.1% random coil, 38.2% ± 1.1% β‐turn/α‐helix). At 2 and 16 h, FBs further evolved to contain significantly higher antiparallel β‐sheet rich structure with a significant decrease in β‐turn/α‐helix (2 h: 25.8% ± 2.27% parallel β‐sheet, 33.3% ± 5.8% antiparallel β‐sheet, 2.6% ± 0.1% random coil, 38.2% ± 1.1% β‐turn/α‐helix; 16 h: 24.4% ± 2.5% parallel β‐sheet, 39.1% ± 6.3% antiparallel β‐sheet, 2.5% ± 0.1% random coil, 34.0% ± 8.3% β‐turn/α‐helix). This demonstrates that DPPC templates hIAPP FBs with greater amounts of β‐turn/α‐helix in early stages of aggregation, which evolve into antiparallel β‐sheet rich conformations over time.

**FIGURE 5:**
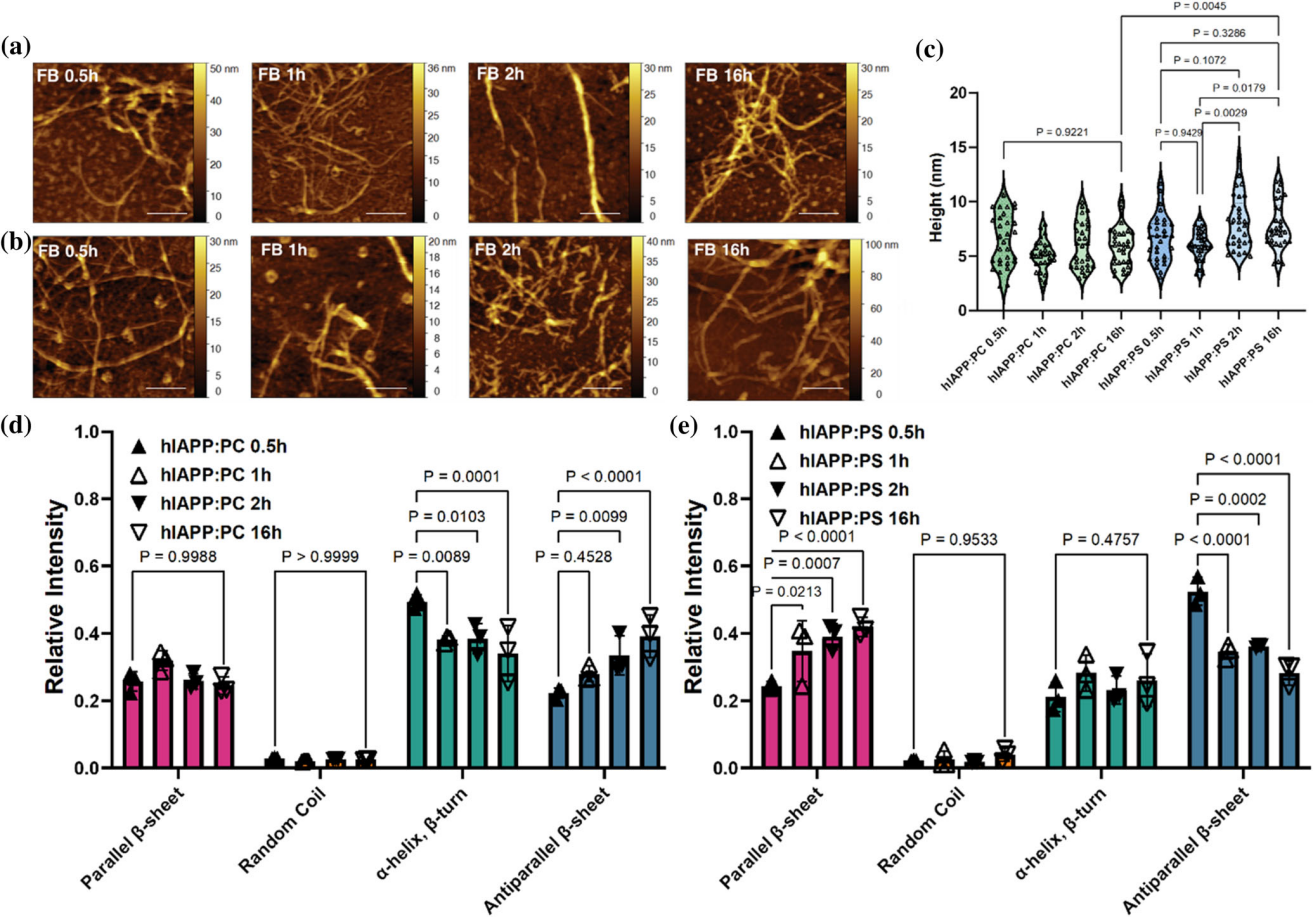
Representative 2.0 × 2.0 μm AFM images of hIAPP fibrils (FBs) formed in the presence of (a) DPPC or (b) DPPS LUVs at a 1:1 protein:lipid molar ratio after 0.5, 1, 2, or 16 h of aggregation. (b) Quantification of profile height from *n* = 30 FBs per condition. Quantification of relative secondary structure content Gaussian peak fitting of *n* = 3 FB AFM‐IR spectra per condition. Image scale bars = 500 nm. Statistical testing performed using two‐way ANOVA with Tukey's post hoc test.

Conversely, hIAPP:PS FBs formed after 0.5 h had a predominantly antiparallel β‐sheet secondary structure (24.3% ± 1.4% parallel β‐sheet, 52.4% ± 4.2% antiparallel β‐sheet, 2.5% ± 2.5% random coil, 21.1% ± 4.3% β‐turn/α‐helix) with no significant difference in FB thickness compared to hIAPP:PC (Figure 5b,c,e). At 1 h of aggregation, hIAPP:PS FBs evolved to contain significantly greater parallel β‐sheet content (34.7% ± 9.0%) and significantly less antiparallel β‐sheet (34.6% ± 2.2%). By 2 and 16 h of aggregation, hIAPP:PS FBs evolved to significantly more parallel β‐sheet rich conformations (39.0% ± 3.9% and 41.9% ± 2.8%, respectively) and significantly less antiparallel β‐sheet (36.1% ± 0.5% and 28.1% ± 3.5%, respectively). Additionally, while the presence of DPPC yielded no significant effect on FB thickness over time, late‐stage DPPS templated FBs to be significantly thicker compared to both hIAPP:PC FBs and early‐stage hIAPP:DPPS FBs. Comparison of PC‐ and PS‐templated FB evolution therefore shows that while both lipid environments ultimately support β‐sheet rich FBs, hIAPP aggregated in the presence of DPPS yields a faster and more pronounced enrichment of parallel β‐sheet content in FBs, while DPPC templates more antiparallel β‐sheet rich FBs over time. Together, this demonstrates that the lipid environment does not only catalyze hIAPP FB formation but also scaffolds specific structural polymorphs depending on the chemical properties of the lipid environment.

Our results also show that lipids dictate both the dimension of the assembled cross‐section and whether parallel or antiparallel β‐sheet architecture dominates the final fibril polymorph. In a lipid‐free environment, fibrils undergo unidirectional parallelization; their dimensions range from 8.38±2.89nm at 1 h to 9.82±2.92nm at 48 h while their initial parallel β‐sheet content (~65% parallel and ~35% antiparallel β‐sheet at 1 h) rises to an overwhelmingly dominant ~82% parallel β‐sheet (<2% disordered) at 48 h (Warren et al., 2026). In contrast, DPPC templates fibrils that maintain invariant height over time (spanning 6.49±2.42nm at 0.5 h to 6.21±1.83nm at 16 h) and undergo antiparallel β‐sheet enrichment, starting with high α/β‐turn content (49.3%) that steadily declines as antiparallel β‐sheet rises to 39.1% by 16 h with parallel β‐sheet held constant between 25% and 31%. DPPS induces an opposite polymorph trajectory accompanied by significant thickening, growing from 6.86±2.22nm at 0.5 h to 8.53±2.80nm at 16 h; these fibrils initially display high antiparallel β‐sheet content (52.4% antiparallel vs. 24.3% parallel β‐sheet at 0.5 h) before rapidly inverting into parallel β‐sheet–dominated assemblies by 16 h (41.9% parallel β‐sheet and 28.1% antiparallel β‐sheet).

#### 2.2.3 Evolution of round oligomers

When formed in the presence of DPPC, hIAPP ROs were comprised of primarily random coil and β‐sheet secondary structure (25.5% ± 8.2% parallel β‐sheet, 29.6% ± 4.0% anti‐parallel β‐sheet, 41.4% ± 6.7% random coil, and 3.6% ± 1.1% β‐turn/α‐helix) (Figure 6a,d). Additionally, hIAPP:PC ROs were observed to be 10.5 ± 4.0 nm thick, which did not significantly change over the time‐resolved evolution of the aggregates (Figure 6c). Although we observed no significant changes in the secondary structure of DPPC‐templated ROs after 1 h of aggregation, those formed after 2 h evolved to contain significantly lower amounts of random coiling (9.0% ± 8.0%) and significantly greater amounts of β‐turn/α‐helix (41.7% ± 8.5%). After 16 h of aggregation, the amount of random coiling was further reduced (2.6% ± 0.1%) and a significant increase in anti‐parallel β‐sheet was observed (53.6% ± 5.6%). These results demonstrate that in the presence of DPPC, ROs reorganize from disordered to β‐sheet and β‐turn rich over time, approaching a fibril‐like secondary structure while remaining morphologically round.

**FIGURE 6:**
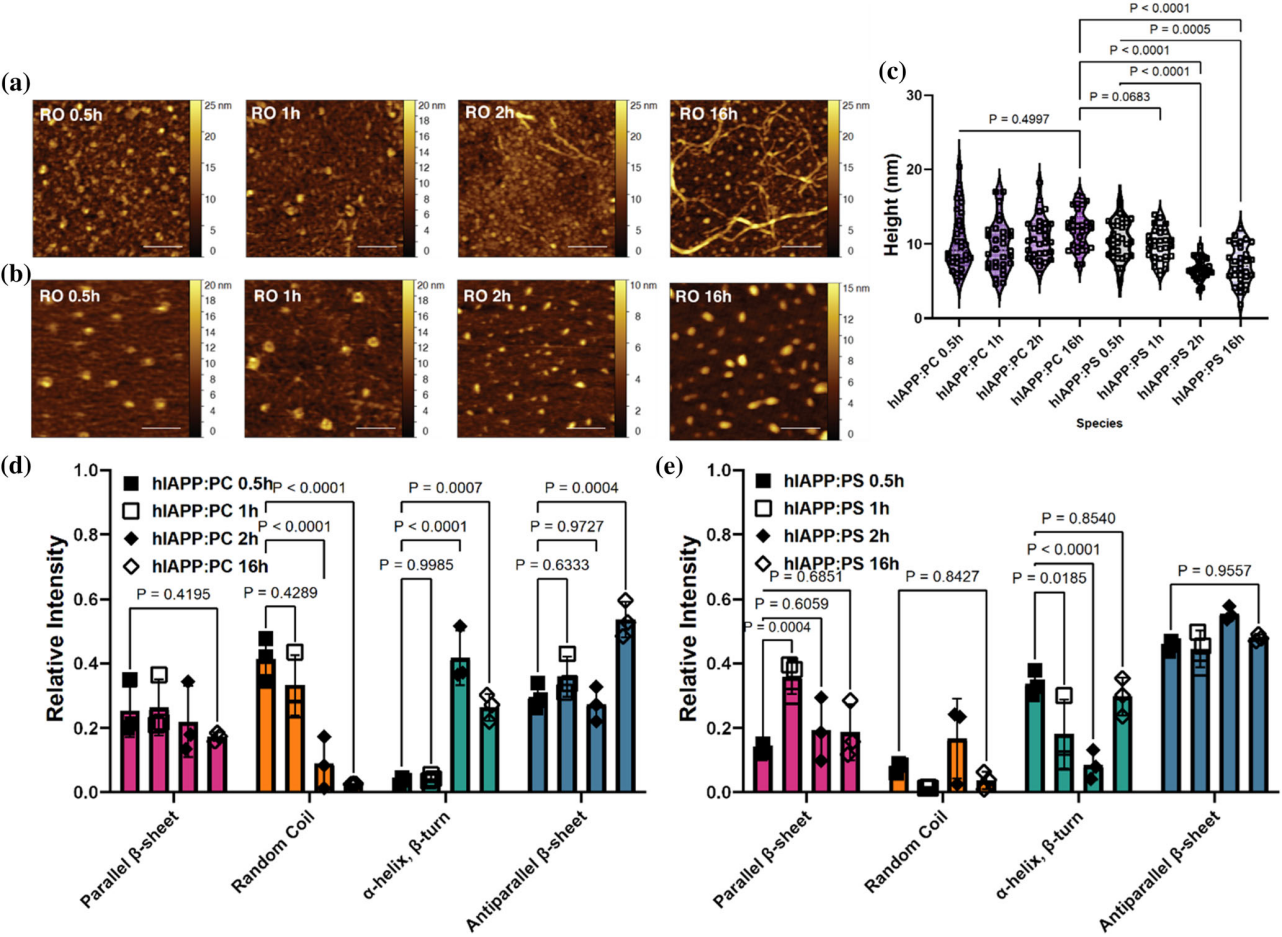
Representative 2.0 × 2.0 μm AFM images of hIAPP round oligomers (ROs) formed in the presence of (a) DPPC or (b) DPPS LUVs at a 1:1 protein:lipid molar ratio after 0.5, 1, 2, or 16 h of aggregation. (b) Quantification of profile height from *n* = 30 ROs per condition. Quantification of relative secondary structure content Gaussian peak fitting of *n* = 3 RO AFM‐IR spectra per condition. Image scale bars = 500 nm. Statistical testing performed using two‐way ANOVA with Tukey's post hoc test.

ROs formed in the presence of DPPS also exhibited time‐dependent morphological and structural evolution. After 0.5 h of aggregation, hIAPP:PS ROs were comprised of a predominantly anti‐parallel β‐sheet structure (13.2% ± 1.7% parallel β‐sheet, 45.4% ± 1.5% anti‐parallel β‐sheet, 7.8% ± 1.6% random coil, and 33.7% ± 3.9% β‐turn/α‐helix) and a comparable thickness to hIAPP:PC ROs (10.43 ± 2.5 nm) (Figure 6b,c,e). After 1 h of aggregation, we observed a shift in PS‐template RO structure, with significantly greater parallel β‐sheet content (35.9% ± 5.3%) and significantly lower β‐turn/α‐helix (18.2% ± 10.6%). After 2 h, the β‐turn/α‐helix content was further reduced in such ROs (8.4% ± 4.5%), and the thickness was significantly reduced (6.7 ± 1.4 nm). Surprisingly, however, hIAPP:PS ROs formed after 16 h were structurally similar to those formed after 0.5 h, although the thickness was significantly lower (7.2 ± 2.6 nm). Together these observations indicate that ROs formed in the presence of PS are more dynamic and transient than those formed in the presence of PC. Additionally, in both DPPC and DPPS, ROs progressively evolve toward ordered β‐sheet/β‐turn aggregates, but the trajectory and final makeup of parallel versus antiparallel β‐sheet structures are lipid dependent.

Thus, lipid environments fundamentally alter both structural evolution trajectories and morphological stability of ROs. Under lipid‐free conditions, ROs undergo gradual, continuous compaction from 14.26±2.42nm at 1 h to 9.95±2.48nm at 24 h, accompanied by a structural shift from highly organized antiparallel β‐sheet at 1 h (~75% antiparallel β‐sheet and ~ 25% parallel β‐sheet) into predominantly disordered states by 4 h (~70% disordered) and 24 h (~60% disordered) (Warren et al., 2026). In the presence of DPPC, this structural trajectory is reversed: ROs begin predominantly disordered at 0.5 h (41.4% random coil, 29.6% antiparallel β‐sheet, and 25.5% parallel β‐sheet) and progressively reorganize into an ordered antiparallel β‐sheet‐rich assembly by 16 h (53.6% antiparallel β‐sheet, 41.7% α/β‐turn at 2 h, and only 2.6% random coil), all while maintaining a remarkably constant height of ~10–12 nm without significant shrinkage. In the presence of DPPS, ROs exhibit marked physical thinning from 10.43±2.5nm at 0.5 h down to 7.2±2.6nm at 16 h alongside stochastic secondary structure fluctuations; they begin antiparallel‐rich (45.4% antiparallel β‐sheet and 33.7% α/β‐turn), temporarily enrich in parallel β‐sheet at 1 h (35.9%), and ultimately consolidate into an antiparallel β‐sheet–dominated form by 16 h (48.4% antiparallel β‐sheet, 30.1% α/β‐turn, and 17.8% parallel β‐sheet).

## 3 DISCUSSION

In the presented study, we demonstrate that the presence of LUVs comprised of two chemically distinct phospholipids, DPPC and DPPS, uniquely alters the early stages of hIAPP aggregation. Utilizing AFM, we observed three morphologically distinct hIAPP aggregate populations, DOs, FBs, and ROs, consistent with what was observed by our group for hIAPP aggregated in the absence of lipids (Warren et al., 2026). Additionally, this previous work identified DOs as transient, on‐path oligomers which dissipated in later stages of aggregation, while ROs were identified as persistent, off‐path oligomers. The data presented here is in good agreement with these previous results, demonstrating that neither DPPC nor DPPS qualitatively disrupt the aggregate classes formed nor their overall progression during aggregation. Additionally, we observed that DO populations were no longer present after 1 h of aggregation in the presence of both DPPC or DPPS LUVs, while in the lipid‐free environment DOs were present for up to 4 h of aggregation. This is in good agreement with Sitton and colleagues, who found that both DPPC and DPPS significantly accelerated hIAPP aggregation (Sitton et al., 2025). Together, these results indicate that lipids not only accelerate hIAPP aggregation but also alter the kinetics of DO formation and evolution.

When comparing results of hIAPP DOs formed in the presence of DPPC or DPPS, we also observe that such aggregates evolve differently. Specifically, DOs formed in the presence of PC contained predominantly parallel β‐sheet and random coiling conformations after 0.5 h of aggregation, while such DOs formed after 1 h had reduced random coiling and greater β‐turn/α‐helix. Conversely, PS‐templated DOs evolved from an antiparallel β‐sheet and β‐turn/α‐helix dominated conformation at 0.5 h to a state with reduced β‐turn/α‐helix and increased random coiling. Additionally, the presence of DPPC and DPPS during aggregation yielded DOs with two distinct structures within the same time point, with PC favoring parallel β‐sheet formation and PS favoring antiparallel β‐sheet. This is consistent with findings by Zhaliazka and coworkers that demonstrated that lipids uniquely alter the structure of amyloid oligomers formed by a peptide of similar size and amyloidogenicity, amyloid β (Aβ) (Zhaliazka et al., 2023). Together, these results indicate that the lipid environment present during hIAPP aggregation uniquely alters both the kinetics of amyloid formation and the structure of the amyloids formed.

A recent study by Wilkinson and colleagues reported that hIAPP FBs continuously evolve over time, yielding distinct FB populations as individual polymorphs interact (Wilkinson et al., 2023). Consistent with these findings, we observed that hIAPP FBs structurally evolve, and this evolution is determined by the chemical properties of the lipid environment. Specifically, FBs formed in the presence of DPPC evolve to contain less β‐turn/α‐helix and more antiparallel β‐sheet over time, while PS‐templated FBs progressively increased in parallel β‐sheet content and decreased in antiparallel β‐sheet. This is consistent with a recent solid state NMR study from Mithu and coworkers who found that lipids uniquely change the hIAPP peptide backbone packing individual β‐strands in FBs (Mithu et al., 2025). Additionally, using AFM‐IR, Sitton and coworkers found that DPPS templated FBs with significantly greater parallel β‐sheet content in comparison to lipid‐free FBs (Sitton et al., 2025). These results indicate that the lipid environment present during hIAPP aggregation determines the structural evolution of hIAPP FBs.

The structural evolution of FBs was previously observed by Warren and coworkers to correlate with that of DOs before DOs dissipate, leading to the conclusion that DOs are on‐path oligomers preceding FB formation (Warren et al., 2026). In good agreement with these findings, we observed that the secondary structure of DOs correlates well with the structural evolution of FBs. DOs formed in the presence of DPPC contained predominantly parallel β‐sheet before dissipating, while hIAPP:PS DOs contained predominantly antiparallel β‐sheet. This aligns well with the structural characteristics of early‐stage FBs formed in each respective condition, which evolve based on the specific lipid environment. In contrast, while ROs do evolve uniquely based upon the lipid environment, this evolution is more stochastic and ROs remain persistent alongside FBs. Thus, we conclude that hIAPP DOs are on‐path oligomers that evolve into FBs, while ROs are persistent, off‐path oligomeric species. In addition, the structural evolution of these individual species is determined by the specific lipid environment present during aggregation (Figure 7).

**FIGURE 7:**
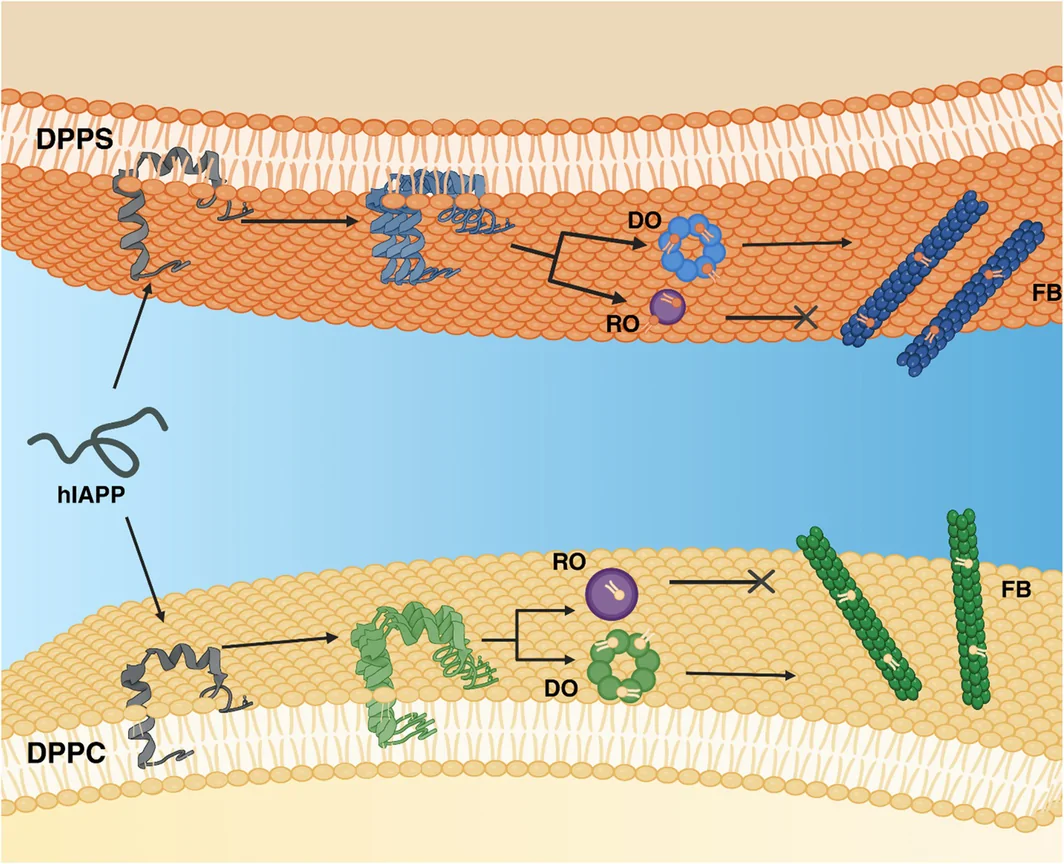
Proposed model by which the lipid environment alters the evolution of hIAPP donut oligomers (DOs), fibrils (FBs), and round oligomers (ROs).

A notable limitation of the present study is the use of monolipid large unilamellar vesicles composed strictly of either DPPC or DPPS as model membrane interfaces. While these simplified single‐component systems are essential for isolating the specific electrostatic contributions of zwitterionic versus anionic headgroups during early‐stage oligomer assembly, native pancreatic β‐cell plasma membranes are remarkably complex. The physiological lipid bilayer comprises asymmetric distributions of diverse phospholipid classes, including phosphatidylethanolamine, phosphatidylinositol, and sphingomyelin, as well as high concentrations of cholesterol. Such multi‐component compositions create heterogeneous lipid rafts, modulate bilayer fluidity, and introduce localized packing defects and surface curvature that can critically influence peptide insertion and oligomeric nucleation. Consequently, the distinct templating pathways observed here for donut and round oligomers in pure DPPC or DPPS systems may be modulated in vivo by cooperative interactions between coexisting lipid species. Future investigations utilizing physiologically relevant lipid mixtures that replicate β‐cell membrane asymmetry and phase behavior will be critical to determine how native membrane heterogeneity coordinates the competition between on‐path and off‐path hIAPP aggregation cascades.

Sample deposition and subsequent ambient drying on gold‐coated substrates can potentially induce capillary forces that flatten soft protein assemblies, typically yielding measured aggregate heights slightly lower than those observed in native liquid environments. Furthermore, substrate wetting and rapid dehydration could conceivably induce minor conformational rearrangements, particularly within flexible, solvent‐exposed random‐coil or turn segments. However, because all aliquots across the DPPC, DPPS, and lipid‐free time points were prepared using identical incubation periods, deposition volumes, and drying protocols on the same substrate batch, any surface‐induced perturbation remains systematic and uniform across conditions. Consequently, the distinct polymorphic differences and time‐dependent secondary structure trajectories reported here reflect genuine lipid‐templated remodeling rather than sample preparation artifacts.

## 4 MATERIALS AND METHODS

### 4.1 Protein preparation

Synthetic hIAPP (1–37) was purchased from Eurogentec as a lyophilized powder with an amidated C‐terminal and a disulfide bridge between cysteine 2 and 7 (1 mg, >95% purity). The powdered protein was dissolved in pure 1,1,1,6,6,6‐hexafluoroisopropanol at a concentration of 1 mg/mL and allowed to incubate for 24 h at 4°C. The solution was then syringe filtered through a 0.22 μm filter, aliquoted, and dried under dry nitrogen gas until a completely dried film was formed. The dried protein was freeze dried at −80°C on dry ice for 48 h and stored at −20°C for no more than 30 days.

### 4.2 Liposome preparation

Powdered lipids were purchased from Avanti Polar Lipids. Lipids were dissolved in 100% chloroform, dried under pressurized air, and freeze‐dried at −80°C for 24 h. Warmed PBS was used to dissolve the frozen lipids at a concentration of 400 μM. The lipid solutions were then flash frozen in liquid nitrogen and thawed repeatedly (8×) until they were extruded through a 100 nm membrane. The generated vesicles' sizes were confirmed with dynamic light scattering using a DynoPro Nanostar II.

### 4.3 Protein aggregation

The dried protein films were allowed to thaw at room temperature before being placed on ice. The thawed protein samples were suspended in chilled sterile Milli‐Q water to reach a protein concentration of 100 μM. The protein solution and pre‐chilled lipid solutions were added to sterile microcentrifuge tubes with chilled 1× PBS to make equal lipid and protein concentrations of 40 μM. The solutions were allowed to aggregate with no agitation at 37°C. Aggregation time points were collected directly from the microcentrifuge tubes and immediately deposited at their respective times for AFM imaging and AFM‐IR spectral analysis.

### 4.4 Protein deposition

At appropriate time points, 3.5 μL of protein solution was removed from the aggregation setup and immediately deposited onto a gold‐coated silica substrate that had been washed with DI water and dried under compressed air. The protein sample was allowed to dry ambiently for exactly 15 min, and a micropipette was used to remove any excess liquid from the substrate. The remaining liquid was allowed to dry completely, then was gently washed with DI water and dried under pressurized air. The deposited samples were stored at room temperature in a closed petri dish.

### 4.5 AFM and AFM‐IR

AFM and AFM‐IR analysis was conducted on a Bruker Nano‐IR3 system. BudgetSensors ContGB‐G probes were used to collect both height and IR spectral data. The probes were optimized using a polymethyl methacrylate (PMMA) standard. Due to an instrumental chip‐to‐chip transition falling in the Amide I window, the probe was also optimized on late‐stage hIAPP fibrils at 1630 cm^−1^. To minimize interference from the chip‐to‐chip transition in data processing, the spectra from 1648 to 1652 cm^−1^ were zapped. The AFM probe was reoptimized between respective lipid samples. Protein samples were scanned at 0.7 Hz and at a resolution of 256 × 256 pts. Thirty IR spectra were collected per time point per discovered species for each lipid condition.

### 4.6 AFM processing and height data analysis

AFM images and heights were processed using Gwyddion software. Images were imported as .AXZ files and processed using the following functions: Facet level, Remove polynomial background (*n* = 1), Fit lines (median of differences), Remove scars. Height data for fibrillar species was collected perpendicular to the fibril axis. Fibrillar species that appeared to be stacked were avoided. Height data for donut‐like species was collected from the depression in the center of the oligomer to its outside. Because the fibril and oligomer populations were so heterogeneous, 30 heights were collected in multiple locations to best represent their entire populations.

### 4.7 IR spectral fitting

IR data was normalized using mean normalization and double smoothed using Savitzky–Golay filtering with a polynomial degree of 1 and window of 9. The IR spectra set within each species were randomized and divided into three groups of 10 spectra. These groups were then averaged and imported into GRAMS AI software to be peak fit using a Gaussian function. The spectra were baselined before peak fitting and areas were assigned to each generated peak within the Amide I region. We assigned the following peak windows to respective secondary structures: parallel β‐sheet (1610–1640 cm^−1^), random coil (1640–1655 cm^−1^), α‐helix/β‐turn (1655–1675 cm^−1^), and antiparallel β‐sheet (1675–1700 cm^−1^).

## AUTHOR CONTRIBUTIONS

**Daniel Warren:** Conceptualization; investigation; methodology; visualization; writing – original draft; writing – review and editing. **Jadon Sitton:** Investigation; writing – review and editing; writing – original draft. **Joshua Skrehot:** Investigation; writing – review and editing. **Dmitry Kurouski:** Conceptualization; funding acquisition; writing – original draft; writing – review and editing; project administration; resources; supervision.

## CONFLICT OF INTEREST STATEMENT

The authors declare no conflicts of interest.

## Supporting information


**Figure S1.** AFM images acquired at indicated timepoints of hIAPP aggregation in the presence of (a) DPPC or (b) DPPS.

## Data Availability

The data that support the findings of this study are available from the corresponding author upon reasonable request.

## References

[pro70867-bib-0001] Asthana S , Mallick B , Alexandrescu AT , Jha S . IAPP in type II diabetes: basic research on structure, molecular interactions, and disease mechanisms suggests potential intervention strategies. Biochim Biophys Acta Biomembr. 2018;1860(9):1765–1782. 10.1016/j.bbamem.2018.02.020 29518374

[pro70867-bib-0002] Banerjee S , Ghosh A . Structurally distinct polymorphs of tau aggregates revealed by nanoscale infrared spectroscopy. J Phys Chem Lett. 2021;12(45):11035–11041. 10.1021/acs.jpclett.1c02660 PMC8967399 34747175

[pro70867-bib-0003] Brender JR , Salamekh S , Ramamoorthy A . Membrane disruption and early events in the aggregation of the diabetes related peptide IAPP from a molecular perspective. Acc Chem Res. 2012;45(3):454–462. 10.1021/ar200189b PMC3272313 21942864

[pro70867-bib-0004] Centrone A . Infrared imaging and spectroscopy beyond the diffraction limit. Annu Rev Anal Chem. 2015;8(1):101–126. 10.1146/annurev-anchem-071114-040435 26001952

[pro70867-bib-0005] Collaborators, G. B. D. D . Global, regional, and national burden of diabetes from 1990 to 2021, with projections of prevalence to 2050: a systematic analysis for the global burden of disease study 2021. Lancet. 2023;402(10397):203–234. 10.1016/S0140-6736(23)01301-6 PMC10364581 37356446

[pro70867-bib-0006] Dazzi A , Prater CB . AFM‐IR: technology and applications in nanoscale infrared spectroscopy and chemical imaging. Chem Rev. 2017;117(7):5146–5173. 10.1021/acs.chemrev.6b00448 27958707

[pro70867-bib-0007] Dazzi A , Prater CB , Hu QC , Chase DB , Rabolt JF , Marcott C . AFM‐IR: combining atomic force microscopy and infrared spectroscopy for nanoscale chemical characterization. Appl Spectrosc. 2012;66(12):1365–1384. 10.1366/12-06804 23231899

[pro70867-bib-0008] Dou T , Kurouski D . Phosphatidylcholine and phosphatidylserine uniquely modify the secondary structure of alpha‐Synuclein oligomers formed in their presence at the early stages of protein aggregation. ACS Chem Nerosci. 2022;13(16):2380–2385. 10.1021/acschemneuro.2c00355 PMC10405296 35904551

[pro70867-bib-0009] Giri B , Dey S , Das T , Sarkar M , Banerjee J , Dash SK . Chronic hyperglycemia mediated physiological alteration and metabolic distortion leads to organ dysfunction, infection, cancer progression and other pathophysiological consequences: an update on glucose toxicity. Biomed Pharmacother. 2018;107:306–328. 10.1016/j.biopha.2018.07.157 30098549

[pro70867-bib-0010] Hassan S , White K , Terry C . Linking hIAPP misfolding and aggregation with type 2 diabetes mellitus: a structural perspective. Biosci Rep. 2022;42(5):11297. 10.1042/BSR20211297 PMC9118370 35475576

[pro70867-bib-0011] Kanatsuka A , Kou S , Makino H . IAPP/amylin and beta‐cell failure: implication of the risk factors of type 2 diabetes. Diabetol Int. 2018;9(3):143–157. 10.1007/s13340-018-0347-1 PMC6224910 30603362

[pro70867-bib-0012] Katzenmeyer AM , Aksyuk V , Centrone A . Nanoscale infrared spectroscopy: improving the spectral range of the photothermal induced resonance technique. Anal Chem. 2013;85(4):1972–1979. 10.1021/ac303620y 23363013

[pro70867-bib-0013] Laakso M . Hyperglycemia and cardiovascular disease in type 2 diabetes. Diabetes. 1999;48(5):937–942. 10.2337/diabetes.48.5.937 10331395

[pro70867-bib-0014] Lahiri B , Holland G , Centrone A . Chemical imaging beyond the diffraction limit: experimental validation of the PTIR technique. Small. 2013;9(3):439–445. 10.1002/smll.201200788 23034929

[pro70867-bib-0015] Meng L , Li Y , Liu C , Zhang G , Chen J , Xiong M , et al. Islet amyloid polypeptide triggers alpha‐synuclein pathology in Parkinson's disease. Prog Neurobiol. 2023;226:102462. 10.1016/j.pneurobio.2023.102462 37150314

[pro70867-bib-0016] Mithu VS , Giller K , Nimerovsky E , Overkamp K , Andreas LB , Becker S , et al. The structure of human IAPP fibrils reflects membrane and pH conditions. J Am Chem Soc. 2025;147(32):28943–28954. 10.1021/jacs.5c06971 PMC12356532 40748680

[pro70867-bib-0017] Moreno‐Gonzalez I , Edwards G Iii , Salvadores N , Shahnawaz M , Diaz‐Espinoza R , Soto C . Molecular interaction between type 2 diabetes and Alzheimer's disease through cross‐seeding of protein misfolding. Mol Psychiatry. 2017;22(9):1327–1334. 10.1038/mp.2016.230 PMC5495631 28044060

[pro70867-bib-0018] Mucibabic M , Steneberg P , Lidh E , Straseviciene J , Ziolkowska A , Dahl U , et al. Alpha‐Synuclein promotes IAPP fibril formation in vitro and beta‐cell amyloid formation in vivo in mice. Sci Rep. 2020;10(1):20438. 10.1038/s41598-020-77409-z PMC7686322 33235246

[pro70867-bib-0019] Nanga RP , Brender JR , Vivekanandan S , Ramamoorthy A . Structure and membrane orientation of IAPP in its natively amidated form at physiological pH in a membrane environment. Biochim Biophys Acta. 2011;1808(10):2337–2342. 10.1016/j.bbamem.2011.06.012 PMC3156962 21723249

[pro70867-bib-0020] Pickett D , Purvinsh Y , Skrehot JT , Warren D , Kurouski D . Nanoscale morphological and structural analysis of round and donut oligomers formed by C‐terminal domain of TDP‐43. Phys Chem Chem Phys. 2026;28(27):16459–16463. 10.1039/d6cp01760f PMC13325187 42389895

[pro70867-bib-0021] Ramer G , Ruggeri FS , Levin A , Knowles TPJ , Centrone A . Determination of polypeptide conformation with nanoscale resolution in water. ACS Nano. 2018;12(7):6612–6619. 10.1021/acsnano.8b01425 PMC11404133 29932670

[pro70867-bib-0022] Ruggeri FS , Benedetti F , Knowles TPJ , Lashuel HA , Sekatskii S , Dietler G . Identification and nanomechanical characterization of the fundamental single‐strand protofilaments of amyloid alpha‐synuclein fibrils. Proc Natl Acad Sci U S A. 2018;115(28):7230–7235. 10.1073/pnas.1721220115 PMC6048494 29941606

[pro70867-bib-0023] Ruggeri FS , Flagmeier P , Kumita JR , Meisl G , Chirgadze DY , Bongiovanni MN , et al. The influence of pathogenic mutations in alpha‐Synuclein on biophysical and structural characteristics of amyloid fibrils. ACS Nano. 2020a;14(5):5213–5222. 10.1021/acsnano.9b09676 32159944

[pro70867-bib-0024] Ruggeri FS , Longo G , Faggiano S , Lipiec E , Pastore A , Dietler G . Infrared nanospectroscopy characterization of oligomeric and fibrillar aggregates during amyloid formation. Nat Commun. 2015;6:8831. 10.1038/ncomms8831 PMC4525161 26215704

[pro70867-bib-0025] Ruggeri FS , Mannini B , Schmid R , Vendruscolo M , Knowles TPJ . Single molecule secondary structure determination of proteins through infrared absorption nanospectroscopy. Nat Commun. 2020b;11(1):2945. 10.1038/s41467-020-16728-1 PMC7287102 32522983

[pro70867-bib-0026] Sciacca MF , Lolicato F , Di Mauro G , Milardi D , D'Urso L , Satriano C , et al. The role of cholesterol in driving IAPP‐membrane interactions. Biophys J. 2016;111(1):140–151. 10.1016/j.bpj.2016.05.050 PMC4944530 27410742

[pro70867-bib-0027] Sciacca MF , Lolicato F , Tempra C , Scollo F , Sahoo BR , Watson MD , et al. Lipid‐chaperone hypothesis: a common molecular mechanism of membrane disruption by intrinsically disordered proteins. ACS Chem Nerosci. 2020;11(24):4336–4350. 10.1021/acschemneuro.0c00588 PMC7769131 33269918

[pro70867-bib-0028] Sitton J , Pickett D , Hung A , Elsaigh R , Kurouski D . Fatty acids alter to the toxicity of islet amyloid polypeptide aggregates in a length and saturation dependent manner. iScience. 2026;29(1):114401. 10.1016/j.isci.2025.114401 PMC12796004 41536979

[pro70867-bib-0029] Sitton J , Pickett D , Rodriguez A , Kurouski D . Lipids determine the toxicity of human islet polypeptide aggregates in vivo. J Biol Chem. 2025;301(1):108029. 10.1016/j.jbc.2024.108029 PMC11728924 39615682

[pro70867-bib-0030] Skeby KK , Andersen OJ , Pogorelov TV , Tajkhorshid E , Schiott B . Conformational dynamics of the human islet amyloid polypeptide in a membrane environment: toward the aggregation prone form. Biochemistry. 2016;55(13):2031–2042. 10.1021/acs.biochem.5b00507 PMC5733697 26953503

[pro70867-bib-0031] Skrehot JT , Matveyenka M , Kurouski D . 0N4R tau aggregates producing morphologically different and structurally similar “on‐path” and “off‐path” oligomers. Chem Commun (Camb). 2026;62(58):14602–14605. 10.1039/d6cc02327d PMC13347184 42421538

[pro70867-bib-0032] Soong R , Brender JR , Macdonald PM , Ramamoorthy A . Association of highly compact type II diabetes related islet amyloid polypeptide intermediate species at physiological temperature revealed by diffusion NMR spectroscopy. J Am Chem Soc. 2009;131(20):7079–7085. 10.1021/ja900285z 19405534

[pro70867-bib-0033] Warren D , Sitton J , Kurouski D . Structural and morphological dynamics of “on‐path” and “off‐path” oligomers of human islet amyloid polypeptide. Protein Sci. 2026;35(4):e70523. 10.1002/pro.70523 PMC12953715 41772826

[pro70867-bib-0034] Westermark P , Grimelius L . The pancreatic islet cells in insular amyloidosis in human diabetic and non‐diabetic adults. Acta Pathol Microbiol Scand A. 1973;81(3):291–300. 10.1111/j.1699-0463.1973.tb03538.x 4129056

[pro70867-bib-0035] Wilkinson M , Xu Y , Thacker D , Taylor AIP , Fisher DG , Gallardo RU , et al. Structural evolution of fibril polymorphs during amyloid assembly. Cell. 2023;186(26):5798–5811.e5726. 10.1016/j.cell.2023.11.025 PMC7617692 38134875

[pro70867-bib-0036] Zhaliazka K , Kurouski D . Nanoscale characterization of parallel and antiparallel beta‐sheet amyloid beta 1‐42 aggregates. ACS Chem Nerosci. 2022;13(19):2813–2820. 10.1021/acschemneuro.2c00180 PMC10405294 36122250

[pro70867-bib-0037] Zhaliazka K , Matveyenka M , Kurouski D . Lipids uniquely alter the secondary structure and toxicity of amyloid beta 1‐42 aggregates. FEBS J. 2023;290(12):3203–3220. 10.1111/febs.16738 PMC10389563 36705524

[pro70867-bib-0038] Zhou L , Kurouski D . Structural characterization of individual alpha‐Synuclein oligomers formed at different stages of protein aggregation by atomic force microscopy‐infrared spectroscopy. Anal Chem. 2020;92(10):6806–6810. 10.1021/acs.analchem.0c00593 32347706

