# Supplementary material for: Lipid membrane composition modulates the structural evolution of early‐stage human islet amyloid polypeptide amyloids
Source: Protein Sci. 2026 Nov 5;35(12):e70867. doi: 10.1002/pro.70867 (PMC13740689; doi:10.1002/pro.70867)
Supplement: Supplementary file 1 [file PRO-35-e70867-s001.docx]

**Lipid membrane composition modulates the structural evolution of early-stage human islet amyloid polypeptide amyloids**

Daniel Warren^1^, Jadon Sitton^1^, Joshua Skrehot^1^, and Dmitry Kurouski^1,2*^

^1^Department of Biochemistry and Biophysics, Texas A&M University, College Station, Texas, United States

^2^Department of Biomedical Engineering, Texas A&M University, College Station, Texas, United States

Email: [dkurouski@tamu.edu](mailto:dkurouski@tamu.edu)

*Supplementary Information*

**Figure S1.** AFM images acquired at indicated timepoints of hIAPP aggregation in the presence of A) DPPC or B) DPPS.
